# Biomechanical comparison of subscapularis peel and lesser tuberosity osteotomy for double-row subscapularis repair technique in a cadaveric arthroplasty model

**DOI:** 10.1186/s13018-019-1372-x

**Published:** 2019-11-28

**Authors:** Mandeep S. Virk, Saleh S. Aiyash, Rachel M. Frank, Christopher S. Mellano, Elizabeth F. Shewman, Vincent M. Wang, Anthony A. Romeo

**Affiliations:** 1 Department of Orthopaedic Surgery, Division of Shoulder & Elbow, New York University Langone Health, New York, NY, USA; 2 Department of Orthopaedic Surgery, Rush University Medical Center, 1611 West Harrison Street, Suite 300, Chicago, IL 60612 USA; 3 Rothman Orthopaedics- New York, 176 3rd Ave, New York, NY 10003 USA

**Keywords:** Total shoulder arthroplasty, Biomechanical analysis, Subscapularis tenotomy, Subscapularis peel, Lesser tuberosity osteotomy, Subscapularis failure

## Abstract

**Introduction:**

Management of the subscapularis during shoulder arthroplasty is controversial. The purpose of this study was to compare the biomechanical performance of subscapularis peel (SP) and lesser tuberosity osteotomy (LTO) in a cadaveric model.

**Methods:**

The subscapularis and proximal humerus were dissected from all soft tissues in 21 fresh-frozen human cadaveric shoulders and randomized to undergo SP, LTO, or standard subscapularis tenotomy (ST, control). For SP and LTO, six #5 sutures were passed through eyelets in the implant (on lateral border and through drill holes in bicipital groove [2] and under trunion [4]). Double-row repair was performed using two lateral row transosseous sutures and four medial row sutures through the tendon (SP) or osseotendinous junction (LTO). Biomechanical properties and mode of failure were tested.

**Results:**

There were no significant differences in elongation amplitude, cyclic elongation, or maximum load to failure between the three groups (*P* > 0.05). Mean stiffness was significantly higher in LTO (*P* = 0.009 vs. SP and ST). In the ST group, 7/7 specimens failed at the tendon-suture interface. For SP, 4/7 failed at the tendon-suture interface, one at the suture-bone interface, one fractured around the implant stem, and one at the knots. For LTO, 3/7 failed at the tendon-suture interface, two at the suture-bone interface and two fractured around the implant stem.

**Conclusions:**

In this cadaveric model, subscapularis repair via ST, SP, and LTO techniques was biomechanically equivalent. Additional studies are needed to confirm these findings and determine the influence of biologic healing on healing rates and clinical outcomes.

**Level of evidence:**

N/a, biomechanical laboratory study

## Introduction

The standard deltopectoral approach for total shoulder arthroplasty (TSA) requires compromise of the subscapularis tendon for insertion of prosthetic components, necessitating repair of the tendon at the conclusion of the procedure. Postoperative dysfunction of this repair is an increasingly recognized postoperative complication [2, 5, 7, 14] and complete rupture can result in significantly worsens clinical outcomes [14, 16]. While a number of techniques have been described including subscapularis tenotomy, subscapularis peel and lesser tuberosity osteotomy [6, 9, 11, 19], few studies have tested the biomechanical strengths of these repairs [8, 12, 22, 24]. Knowing which of the most commonly used repair technique is the strongest would inform the shoulder surgeon in his/her attempts to prevent this complication.

In a biomechanical study, Ahmad et al. [1] compared the strength of fixation under cyclic loading between a tendon-to-bone repair and a combined tendon-to-tendon/tendon-to-bone technique. Using six matched pairs of cadaveric shoulders, these authors demonstrated improved pull-out strengths with the combined repair. While informative, these results have not been duplicated elsewhere and do not consider a third commonly used subscapularis repair technique, lesser tuberosity osteotomy, which is popularized and elegantly described by Gerber et al. [6] where the lesser tuberosity is osteotomized and repaired as a single bone/tendon unit. In the lesser tuberosity osteotomy repair technique, the opportunity for exclusive bone-to-bone fixation has been shown to heal reliably in the clinical setting [6, 7, 11] and may offer greater pull-out strength. In another biomechanical study Van Thiel and colleagues [25] evaluated three different methods of subscapularis repair following TSA. The authors compared lesser tuberosity osteotomy, tendon-to-bone repair with bone tunnels (where the subscapularis was first removed periosteally), and finally a combined method of repair. Interestingly, the authors found no significant differences in elongation amplitude, cyclic elongation, or ultimate load to failure among the groups, and concluded that biomechanically, all constructs offered similar outcomes.

In 2012, Lapner and colleagues [11] performed a randomized controlled trial comparing patients undergoing TSA with lesser tuberosity osteotomy to those undergoing TSA with subscapularis peel. The authors found that at 24 months follow-up, there was no significant difference in subscapularis muscle strength (*P* = 0.131) or final subjective outcomes. In the peel group, the subscapularis tendon was peeled off the lesser tuberosity at the intertubercular groove. In the osteotomy group, a 5- to 10-mm-thick and 3-cm-long fragment of the lesser tuberosity was elevated along with the subscapularis insertion as described by Gerber et al. [6]. After implantation of the prosthesis, the tendon was repaired identically in both groups, with three nonabsorbable mattress transosseous sutures passed within the bicipital groove and tied over a mini-plate placed on the lateral aspect of the greater tuberosity.

Recently, a new humeral system for TSA was introduced, and it attempts to use the actual implant to aid in the subscapularis repair. The Univers APEX (Arthrex, Inc., Naples, FL) is a humeral component composed of titanium and consisting of a stem, trunion, inclination block, pins, and screws. The humeral component is implanted with a series of sutures placed through the actual implant. Two of these sutures are subsequently passed through drill holes made in the bicipital groove, while the remaining are passed through the subscapularis with free needles to allow for an anatomic subscapularis repair. Lederman et al [12] evaluated this novel technique when compared to lesser tuberosity osteotomy however no biomechanical study analyzing this suture technique has compared multiple subscapularis repair techniques including tenotomy. The purpose of the proposed study is to compare cyclic loading strength, slippage, and ultimate load-to-failure strengths of three tendon repair techniques (tenotomy, peel, and “sliver” lesser tuberosity osteotomy) in a cadaveric arthroplasty model utilizing the Univers APEX (Arthrex, Inc., Naples, FL) humeral stem.

## Methods

This study was labeled as exempt from our university’s institutional review board. A total of 21 fresh-frozen human cadaveric shoulders with an average age of 60.1 ± 7.9 years were included for the final analysis. Computed tomography (CT) scans were performed prior to specimen dissection and preparation. Specimens with bony lesions were excluded (*n* = 1). Bone mineral density (BMD) was calculated from CT scans of the proximal humerus as described previously [22, 25].

**Fig. 1:**
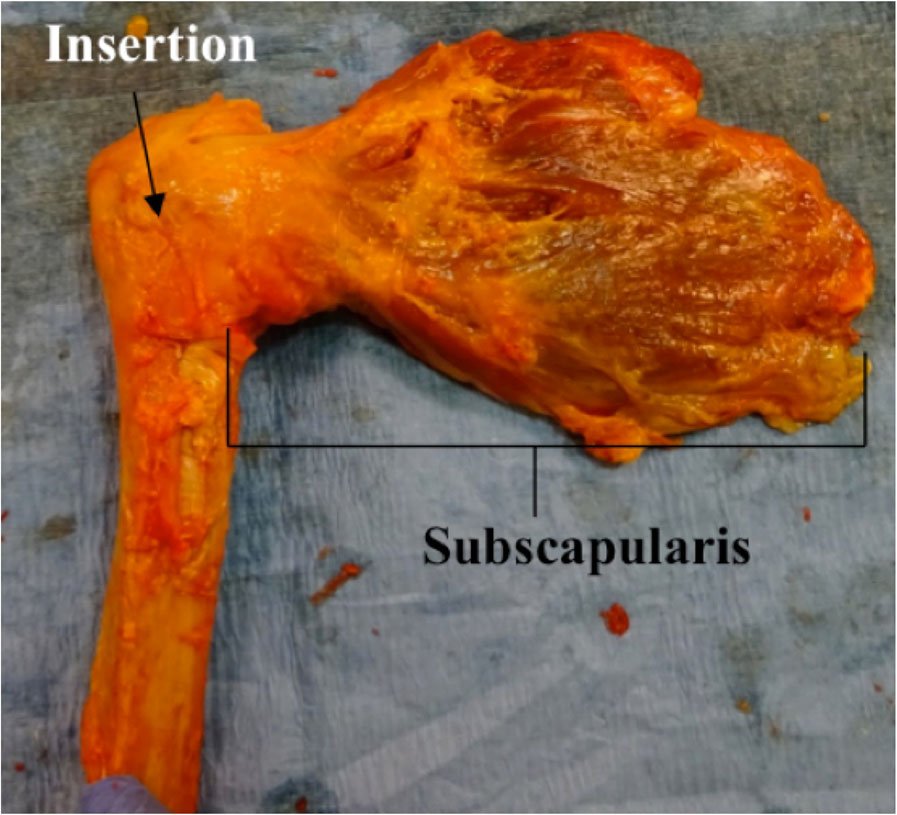
Right shoulder cadaveric specimen demonstrating the dissection utilized to isolate the subscapularis muscle-tendon unit

Following thawing, the soft tissues were dissected to the level of the rotator cuff musculature. Another surgeon performed all dissections and visually inspected specimens for rotator cuff tears. Specimens with partial- or full-thickness rotator cuff tears (subscapularis, supraspinatus, or infraspinatus) were excluded. Eleven shoulders were excluded because of rotator cuff tears (4), bone lesion (1), inadequate fixation (4), and technical errors during biomechanical testing (2). The subscapularis was reflected off the scapula, and the humerus was detached from the glenoid with the subscapularis tendon intact. All tendons except the subscapularis and 1 cm of the anterior leading edge of the supraspinatus insertion were released sharply from their humeral insertions (Fig. 1). The specimens were subsequently randomized into three groups based on repair technique:

I. Subscapularis tenotomy (ST; *N* = 7); control
II. Subscapularis peel (SP; *N* = 7)
III. Lesser tuberosity osteotomy (LTO; *N* = 7)

Subscapularis release from the humerus was performed based on the assigned repair technique. For specimens in group I, the tendon was tenotomized 1 cm medial to its insertion [25].

For group II, the tendon was peeled directly off its footprint as published previously [11]. In group III, the lesser tuberosity was osteotomized as described by Gerber et al. [6].

### Humerus preparation and subscapularis repair

For each specimen, humeral preparation proceeded as for standard anatomic TSA. The natural inclination and version of the humeral head was marked with an indelible marker. A wide-blade oscillating saw was used to perform the humeral head osteotomy along the native retroversion and inclination followed by canal reaming and broaching. Trialing was performed to determine the optimum press fit of the humeral component. At this point, the type of repair determined the remaining part of the procedure.

In the ST group, three 2-mm drill holes were made through the lateral part of the subscapularis footprint (equally spaced) and three #5 high strength non-reabsorbable sutures (Arthrex Inc, Naples, FL) were passed. The implant was placed into the canal, and the medial ends of the sutures were passed through the subscapularis tendon in a Mason-Allen configuration. A rotator interval suture was passed through the anterior leading edge of the supraspinatus tendon and then through the upper rolled edge of the subscapularis tendon using a #2 high strength non-reabsorbable suture (Arthrex Inc, Naples, FL) and tied first to get the subscapularis tendon reduced anatomically. The medial and lateral ends of the three sutures passed through the subscapularis tendon were tied next. After this, three #5 high strength non-reabsorbable sutures (Arthrex Inc, Naples, FL) were passed in simple configuration through the lateral and medial part of the subscapularis tenotomy and tied. At the conclusion of the repair, there were a total of six sutures (alternating Mason-Allen and simple sutures) across the subscapularis tenotomy and one suture across the rotator interval.

**Fig. 2:**
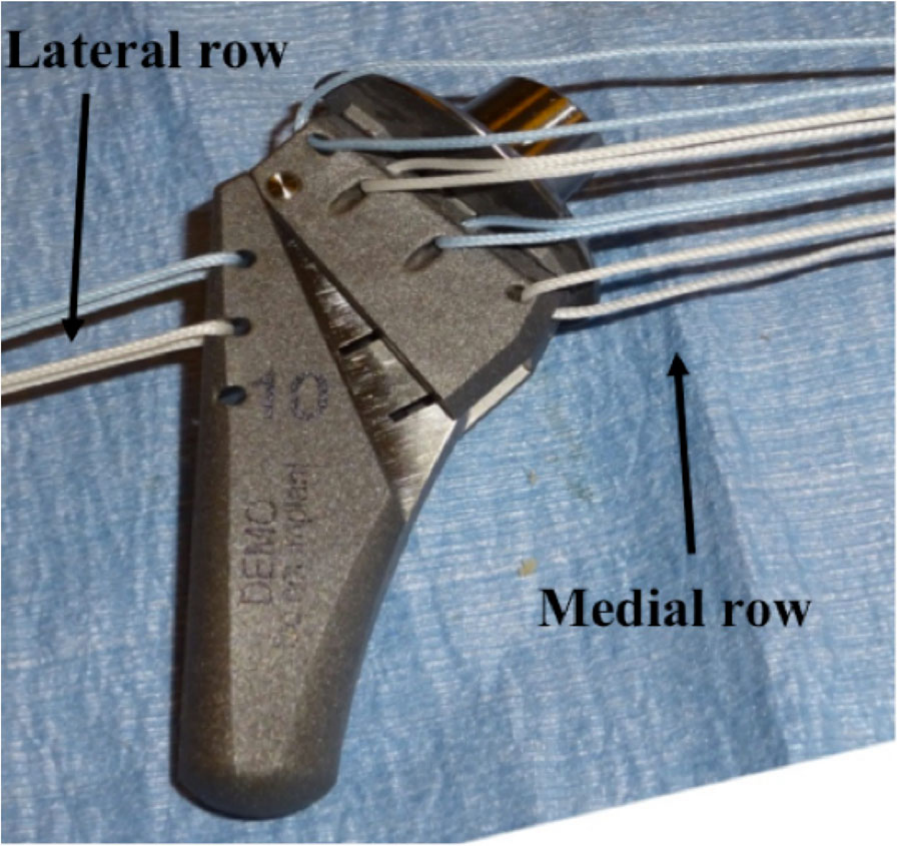
Photograph demonstrating the use of 6 (2 lateral, 4 medial) #5 high strength nonabsorbable sutures placed through the implant prior to implant insertion into the humerus

In the SP and LTO groups, after trialing for stem size, two unicortical drill holes (2 mm) were drilled in the biceps groove. The proximal drill hole was made at the junction of the upper and middle third of the subscapularis footprint, and the distal drill hole was made at the junction of the middle and distal third of the footprint. A short-stemmed humeral stem component (APEX; Arthrex Inc., Naples, FL) was used in a press-fit fashion. Prior to implanting the humeral component, two high strength #5 nonabsorbable (Arthrex Inc., Naples, FL) lateral row sutures (blue and white color) were passed through the eyelets in the proximal lateral part of the body and shuttled through the transosseous holes in the bicipital groove yielding four suture limbs labeled A through D. The four high strength #5 nonabsorbable medial row sutures (Arthrex Inc., Naples, FL) were passed through the four eyelets located under the trunion of the humeral component yielding eight suture limbs labeled 1 through 8 (suture colors are blue (1), white (2, 3), blue (4, 5), white (6, 7) from proximal to distal, Fig. 2). The lateral row suture limbs were passed transosseously through the bicipital groove (A and B through proximal hole; C and D through the distal hole). At this point, all suture strands were held out to tension as the stem was implanted in the humeral canal. The medial row sutures, exiting transosseously at the calcar (limbs 1 through 8) were then passed through the subscapularis tendon (or at the junction tendon to bone in LTO) in a Mason Allen configuration. To complete the subscapularis repair, the sutures were then tied in the following sequence:

A. 1 to A (blue to blue)
B. 8 to D (tiger white to tiger white)
C. 4 to C (tiger white to tiger white)
D. 5 to B (blue to blue)
E. 2 to 3 (blue to tiger white)
F. 6 to 7 (blue to tiger white; these sutures compress the tendon repair to the lesser tuberosity)

**Fig. 3:**
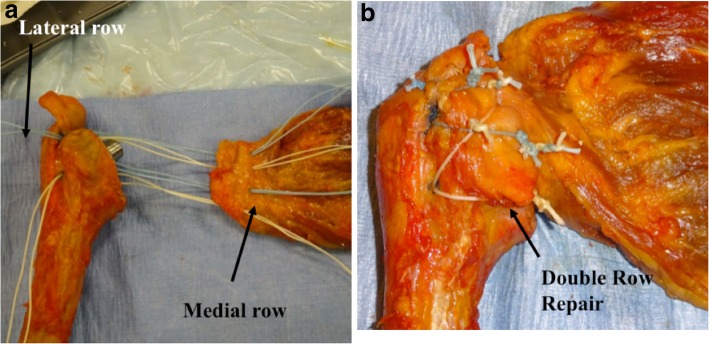
**a** Right shoulder cadaveric specimen demonstrating how the sutures are placed through the subscapularis tendon interface for the SP and LTO repair prior to the sutures being tied down. **b** Final double-row repair construct after all sutures are tied down

In suture-tying process, one lateral row limb is tied to the one medial row limb from each of the four medial row sutures respectively to form a double-row construct (from superior to inferior). The remaining four medial row limbs are tied to each other (top two and bottom two) to eliminate any slack in the repair and create a dynamic compression of the subscapularis tendon or osteotomy fragment over the lesser tuberosity (Fig. 3a, b). The different suture color pattern aids in suture management and avoids mismatch of suture tying. The first four knots between lateral and medial row are tied between similar colors, and the last two knots (for dynamic compression) are tied between different colors in the medial row.

### Biomechanical testing

Following subscapularis repair, the proximal humerus for each specimen was potted within a plastic cylinder using acrylic cement (Isocryl, Lang Dental, Wheeling, IL) and mounted to a custom alignment fixture secured to the base of an electromechanical materials testing system (MTS Insight 5, Eden Prairie, MN). The subscapularis muscle-tendon units were rigidly secured to an aluminum plate with the direction of loading in line with the natural subscapularis tendon vector. The arm was maintained in neutral rotation, abduction, and flexion. The subscapularis muscle belly was fixed in a customized cryogenic clamp. Three rows of three spatial markers were placed, two on either side of the repair construct as well as one on the upper grip. Markers were numbered 1 to 3 from superior to inferior. High-resolution digital imaging equipment was used to visualize the movement of the markers (1000 × 1000 × 48 fps, Imperx IPX-1M48-L, Boca Raton, FL), and digital motion analysis software (Spica Technology Corp, Maui, HI) was used to optically record the marker displacements to assure no slippage at the grip.

**Fig. 4:**
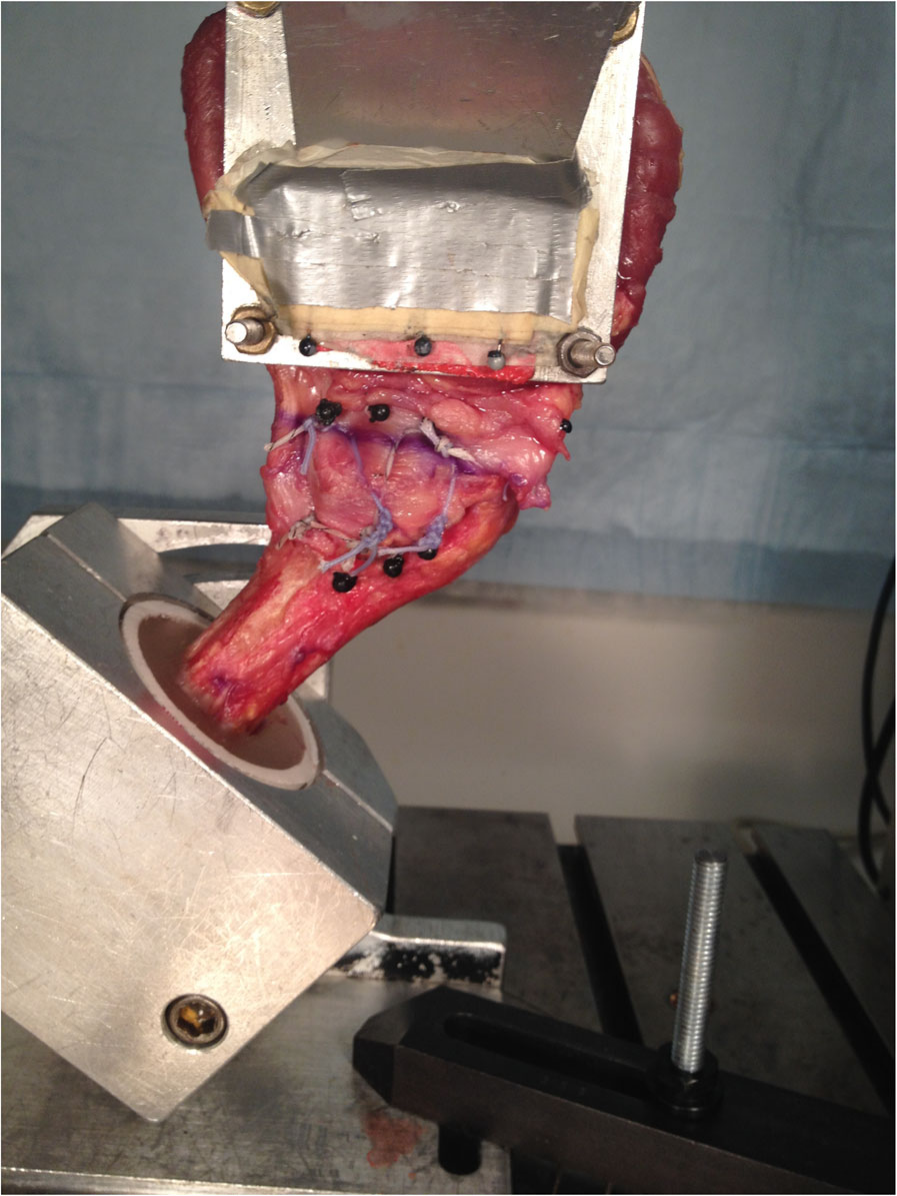
Shoulder arthroplasty cadaveric specimen demonstrating biomechanical testing on the MTS machine

Biomechanical testing (Fig. 4) was then performed via the tensile apparatus, using cyclic loading under force control, with each specimen being maintained in a moist state throughout the testing. To start, each specimen was preloaded to 10 N for 1 min and then loaded for 150 cycles from 10 to 100 N at 0.5 Hz. After cyclic testing, each specimen was pulled to failure at a rate of 1 mm/s. Throughout cyclic and failure testing, load, actuator displacement, and time were recorded synchronously with the optical data using dedicated MTS Test Works software. Construct failure mode was visually classified as occurring within the muscle, tendon, repair site, or bone.

During cyclical loading, repairs were assessed for (1) first cycle excursion, which is peak displacement in the first cycle; (2) cyclical elongation, defined as the increase in segment length from the peak load of the first cycle to the peak load of the final cycle; (3) cyclic creep, defined as peak displacement at the last cycle; and (4) elongation amplitude, defined as the peak-valley measurement of the segment elongation for the final cycle. During pull-to-failure testing, repairs were assessed for (1) maximum load; (2) extension at maximum load; (3) linear stiffness, which was calculated as the steepest slope of the load-displacement curve between initiation of the load-to-fail test; and (4) energy to failure at maximum load.

## Statistical analysis

The anatomical and biomechanical data was reported as mean ± standard error of mean. The data was compared between the two groups using ANOVA test (Graphpad Prism 5®, Graph Pad, LaJolla, CA). The categorical data was compared using chi-square test. The level of significance was set at *P* ≤ 0.05.

## Results

### Demographic data

A total of 21 specimens were included in the analysis. There were 7 specimens per group and the demographic, BMD, and geometric data of subscapularis tendon in the ST, SP, and LTO groups are summarized in Table 1. There were no significant differences between the 3 groups with respect to the mean age and sex distribution. The bone mineral density was not significantly different between the 3 groups (*P* = 0.48). The subscapularis tendon thickness (upper half) and tendon width were not significantly different (*P* = 0.54 and 0.39 respectively) in the 3 groups (Table 1).

**Table 1:** Demographic data of cadaveric specimens

Variable*	Tenotomy	Peel	Lesser tuberosity osteotomy
Number of specimens	7	7	7
Age (year)	61.6 ± 6.3	58 ± 8.8	60.9 ± 8.7
Sex (M:F)	5:2	6:1	4:3
BMD (g/cm^2^)	145.3 ± 35.1	169.7 ± 60.7	168.1 ± 56.9
Subscapularis tendon thickness (mm)	5.5 ± 1.7	6.5 ± 1.6	6.1 ± 1.4

*Mean ± SD, except for the sex proportion

### Cyclical loading and pull-to-failure data

Cyclic testing of repaired specimens in the three groups did not show any significant differences as shown in Table 2 (*P* > 0.05 for all). No statistically significant differences were noted in cyclic creep (*P* = 0.25), first cycle excursion (*P* = 0.52), elongation amplitude (*P* = 0.06), and cyclic elongation (*P* = 0.47) among the three repair techniques.

**Table 2:** Biomechanical outcomes during cyclic and failure testing

Testing	Variable	Tenotomy	Peel	LTO	P value
Cyclic	First cycle excursion (mm)	3.8 ± 0.42	3.4 ± 0.53	3.2 ± 0.13	0.52
	Elongation amplitude (mm)	2.7 ± 0.35	2.4 ± 0.51	2.1 ± 0.29	0.06
	Cyclic elongation (mm)	2.9 ± 0.76	2.5 ± 1.2	2.2 ± 1.2	0.46
	Cyclic creep (mm)	6.7 ± 1.8	5.5 ± 1.8	5.3 ± 1.1	0.25
Failure	Energy to failure (N*mm)	7057 ± 2673	7959 ± 3208	8765 ± 3785	0.62
	Max Load (N)	596.5 ± 88	701 ± 156	750.7 ± 226	0.23
	Stiffness (N/mm)	46.9 ± 8.7	49.4 ± 8.7	63.6 ± 11.2^a^	0.009
	Extension at maximum load (mm)	24 ± 2	25 ± 2.3	23 ± 2.1	0.81

^a^vs. Tenotomy and Peel

Pull-to-failure testing demonstrated significantly higher mean stiffness in the LTO repair group (*P* = 0.009) compared to the ST and SP groups. However, there were no significant differences between the three groups with respect to maximum load (*P* = 0.23), maximum extension (*P* = 0.81), and energy to failure (*P* = 0.62).

### Modes of failure

The modes of failure during pull-to-failure testing are shown in Table 3. All specimens in the ST group eventually failed due to suture pulling through the tendon during load-to-failure testing. In the SP group, four specimens failed due to suture pulling through the tendon, and in one specimen, the knot unraveled. In two specimens in the SP group, the bone fractured at the level of repair pulling the humerus stem out along with the repair. In the osteotomy group, three specimens failed due to suture pulling through the tendon. In two specimens in the LTO group, the bone fractured at the level of repair pulling the humerus stem out along with the repair, and in two remainder specimens, the suture cut through bone.

**Table 3:** Failure mechanisms during load-to-failure testing in three groups

Groups	Failure mechanisms			
	Tendon-suture interface	Knot failure	Suture-bone interface	Fracture around the component
Tenotomy	7	0	0	0
Peel	4	1	0	2
LTO	3	0	2	2

## Discussion

The management of subscapularis tendon in anatomic total shoulder arthroplasty is an ongoing debate. The results of this cadaveric study demonstrate that there is no significant difference between the repair displacement during cyclic loading, maximum load, and load-to-failure mechanical properties between the tenotomy, peel, and osteotomy repair techniques using the Arthrex APEX TSA components. The stiffness of the repair construct was significantly higher in the LTO technique compared to the peel and tenotomy groups.

In this study, we used a double-row subscapularis repair in the peel and osteotomy group. In this technique, four medial row sutures pass through the eyelets of the humeral component before they are passed through the tendon to provide strong anchorage. The two lateral row sutures pass through the eyelets in the stem and exit through the two holes in the bicipital groove. The four medial and lateral row sutures are tied to each other to achieve static compression, and the remaining four medial sutures are tied to each other to achieve dynamic compression across the repair site. Studies have demonstrated that double-row fixation in regard to rotator cuff repairs results in an increased area of contact between the repair footprint and bone. Biomechanical data in a cadaveric model demonstrate that double-row fixation results in increased ultimate failure strength [4]. This study demonstrates that despite using the dynamic compression subscapularis repair technique, we did not find significant differences between the three repair techniques during cyclic biomechanical testing. Load-to-failure testing demonstrated that LTO repair technique has higher stiffness compared to the peel and tenotomy techniques. Other investigators have reported similar results when comparing the tenotomy, peel, and LTO using standard repair techniques [12, 14, 24, 25]. Van Thiel et al. [25] reported no significant differences in cyclic elongation, cyclic amplitude, maximum load, stiffness, and load to failure between the tenotomy, peel, and LTO repair techniques in a cadaveric experiment.

The outcome of total shoulder arthroplasty is adversely affected by postoperative subscapularis insufficiency [15, 16, 21]. Compromise of subscapularis repair can present as a complete insufficiency (failure of repair) or more commonly as loss of function (loss of terminal internal rotation, weak belly press, and poor lift-off) [9, 14, 15, 20, 21]. Clinical and radiographic studies have demonstrated higher healing rates and lower clinical signs of subscapularis dysfunction when using lesser tuberosity osteotomy compared to the tenotomy and/or transosseous repair [3, 10, 11, 17–19]. However, other studies and recent study do not show any significant difference in the outcomes between the three repair techinques [11, 13] . The osteotomy has demonstrated failure due to nonunion, which can occur due to failure of fixation. We hypothesized that improved time equal zero fixation will minimize or prevent this failure of poor fixation. In this technique, we demonstrated that fixation with osteotomy technique had higher stiffness, higher mean energy to failure, and maximum strength although statistical significance was only achieved for the stiffness parameter.

The three techniques, ST, SP, and LTO, heal via three different mechanisms, namely, tendon to tendon, tendon to bone, and bone to bone respectively. However, in this controlled experimental study, only time zero fixation was assessed. Proponents of tendon-to-tendon repair claim that it is a quick and simple repair technique and avoids the possibility of nonunion of osteotomy. However, MRI and USG data has shown a higher failure compared to osteotomy. Fortunately, this compromised subscapularis function has not translated into poor postoperative outcomes [9, 20, 23]. In actual practice, repair techniques using subscapularis suturing like the peel and tenotomy are more common than lesser tuberosity osteotomy [19].

### Strengths

The strengths of this study include the comprehensiveness of the biomechanical testing and the inclusion of three different commonly used techniques of subscapularis tendon repair in total shoulder arthroplasty spanning three different mechanisms of fixation: tendon to tendon, tendon to bone, and bone to bone. All standard forms of biomechanical testing were performed including cyclic testing and pull-to-failure. In addition, these modes of testing were expanded to test first cycle excursion, cyclical elongation, cyclic creep, and elongation amplitude in cyclic testing and maximum load, extension at maximum load, linear stiffness, and energy to failure at maximum load in the pull-to-failure testing. Additionally, this study is the first to test a suturing technique that utilizes suture integration into the humeral stem in the repair of the subscapularis tendon.

### Limitations

One of the known limitations of biomechanical studies is they assess repair integrity at time zero, which has several disadvantages when correlating clinically. These studies are generally performed on cadaveric models and are therefore not appropriate for determining ultimate healing and strength of the repaired tendon. Additionally, they lack chronicity, which is a critical factor in healing rates and clinical outcome. There are advantages to comparing various repair configurations using a time equal zero model including the ability to study failure modality, interface motion, gap formation, and repair surface area, all of which are important factors in a successful repair. There is also a hypothetical concern for increased risk of infection and tissue necrosis with the use of nonabsorbable sutures as described in this paper. Nonabsorbable sutures in conjunction with double-row repairs are often used for rotator cuffs as they are made of strong materials that allow for physiologic loading conditions. The alternative would be to use slow absorbable sutures to lower the risk of infection and tissue necrosis.

## Conclusions

In this cadaveric shoulder arthroplasty model, subscapularis repair via tenotomy, SP, and LTO techniques was biomechanically equivalent with respect to elongation amplitude, cyclic elongation, and maximum load to failure. Additional studies are needed to confirm these biomechanical findings and determine the influence of biologic healing on overall healing rates and clinical outcomes.

## Data Availability

All data generated or analyzed during this study are included in this published article.

## Abbreviations

BMD: Bone mineral density
CT: Computed tomography
LTO: Lesser tuberosity osteotomy
SP: Subscapularis peel
ST: Subscapularis tenotomy
TSA: Total shoulder arthroplasty
